# Conformational heterogeneity coupled with β-fibril formation of a scaffold protein involved in chronic mental illnesses

**DOI:** 10.1038/s41398-021-01765-1

**Published:** 2021-12-17

**Authors:** Abhishek Cukkemane, Nina Becker, Mara Zielinski, Benedikt Frieg, Nils-Alexander Lakomek, Henrike Heise, Gunnar F. Schröder, Dieter Willbold, Oliver H. Weiergräber

**Affiliations:** 1 Institute of Biological Information Processing (IBI-7: Structural Biochemistry), Forschungszentrum Jülich, Jülich, Germany; 2 Institut für Physikalische Biologie, Heinrich Heine University Düsseldorf, Düsseldorf, Germany; 3 Jülich Centre for Structural Biology (JuStruct), Forschungszentrum Jülich, Jülich, Germany; 4 Physics Department, Heinrich Heine University Düsseldorf, Düsseldorf, Germany

**Keywords:** Physiology, Molecular neuroscience, Schizophrenia

## Abstract

Chronic mental illnesses (CMIs) pose a significant challenge to global health due to their complex and poorly understood etiologies and hence, absence of causal therapies. Research of the past two decades has revealed dysfunction of the disrupted in schizophrenia 1 (DISC1) protein as a predisposing factor involved in several psychiatric disorders. DISC1 is a multifaceted protein that serves myriads of functions in mammalian cells, for instance, influencing neuronal development and synapse maintenance. It serves as a scaffold hub forming complexes with a variety (~300) of partners that constitute its interactome. Herein, using combinations of structural and biophysical tools, we demonstrate that the C-region of the DISC1 protein is highly polymorphic, with important consequences for its physiological role. Results from solid-state NMR spectroscopy and electron microscopy indicate that the protein not only forms symmetric oligomers but also gives rise to fibrils closely resembling those found in certain established amyloid proteinopathies. Furthermore, its aggregation as studied by isothermal titration calorimetry (ITC) is an exergonic process, involving a negative enthalpy change that drives the formation of oligomeric (presumably tetrameric) species as well as β-fibrils. We have been able to narrow down the β-core region participating in fibrillization to residues 716–761 of full-length human DISC1. This region is absent in the DISC1^Δ22aa^ splice variant, resulting in reduced association with proteins from the dynein motor complex, viz., NDE-like 1 (NDEL1) and lissencephaly 1 (LIS1), which are crucial during mitosis. By employing surface plasmon resonance, we show that the oligomeric DISC1 C-region has an increased affinity and shows cooperativity in binding to LIS1 and NDEL1, in contrast to the noncooperative binding mode exhibited by the monomeric version. Based on the derived structural models, we propose that the association between the binding partners involves two neighboring subunits of DISC1 C-region oligomers. Altogether, our findings highlight the significance of the DISC1 C-region as a crucial factor governing the balance between its physiological role as a multifunctional scaffold protein and aggregation-related aberrations with potential significance for disease.

## Introduction

Chronic mental illnesses (CMIs), e.g., schizophrenia and recurrent affective disorders, remain enigmatic due to their multifactorial etiology that involves an interplay of various factors including biological, environmental, and social conditions. One of the major biological risk factors that was identified about 20 years ago in a Scottish pedigree with severe psychiatric disorders is disrupted in schizophrenia 1 (DISC1) isoform 1 [1, 2]. A growing wealth of information on the physiological role of DISC1 describes its importance in cellular functions such as proliferation, neuronal development, and synaptogenesis [3–5]. Despite the lack of hits for DISC1 in genome-wide association study (GWAS) screens [6], there is evidence of the role of DISC1 mutations that have been shown to be heritable in the pathology of schizophrenia and related CMIs [1, 2, 7]. While the GWAS studies have been designed to identify genetic factors associated with phenotypic traits of a disease, it has underperformed for schizophrenia studies at the population level mainly due to identifying common variants. It does not account for mutations that are rare, such as those observed in DISC1 [8, 9], and the ones that are associated with altered post-translational modifications, misassembly, and aggregation [10–12]. Like aberrant species found in other proteinopathies among the neurodegenerative and prion amyloid diseases, DISC1 aggregates have been demonstrated to spread between cells in in vitro experiments [13], albeit without causing cell death. The protein plays the pivotal role of a scaffold by regulating the activity of various enzymes and other proteins, many of which are of clinical and therapeutic relevance; in fact, its interactome includes more than 300 different partners [14, 15]. Therefore, high-resolution 3D structures of the DISC1 protein and its complexes with relevant risk factors are pertinent in comprehending its physiological significance.

**Fig. 1:**
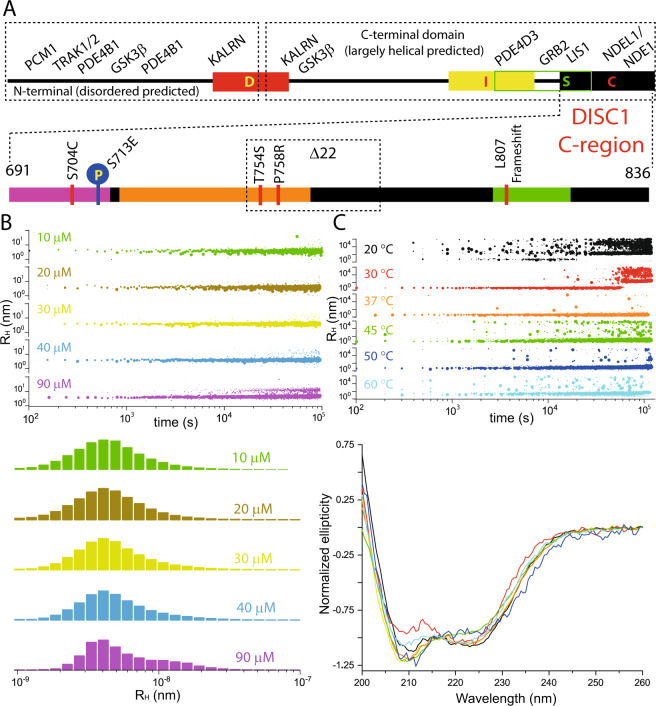
Size distribution and secondary structure composition of DISC1 C-region oligomers. **A** Pictorial representation of the primary structure of the DISC1; the D, I, S and C regions and the potential binding-partner interaction sites are annotated. For more details, the reader is referred to reviews in references [3–5, 8, 9, and 14]. The lower panel shows the C-region with the V_H_H B5 nanobody-binding site (magenta), the (pseudo)repeat sequence (orange), and the C-terminal NDEL1-binding site (green); the region missing in the Δ22 variant is indicated by a dotted box. The same color-coding scheme is also used in the structural model in Fig. 4D. Post-translational modification and mutation sites have been annotated for the C-region. **B** DLS measurements on the His_6_-tagged version at 37 °C, using various concentrations: 10 µM (green, apparent hydrodynamic radius (R_H_): 4.1 nm ± 0.7 nm, calculated molecular mass (M): 81.5 kDa), 20 µM (brown, R_H_: 4 ± 0.6 nm, M: 81.3 kDa), 30 µM (yellow, R_H_: 4.1 ± 0.5 nm, M: 84 kDa), 40 µM (sky blue, R_H_: 4.4 ± 0.5 nm, M: 87 kDa; R_H_: 17.2 ± 3.6 nm, M: 2.3 MDa; R_H_: 18.9 ± 4.4 nm, M: 2.8 MDa), and 90 µM (magenta, R_H_: 4.1 ± 0.6 nm, M: 85.7 kDa; R_H_: 13 ± 2.5 nm, M: 1.2 MDa). The estimated molecular mass of the smallest species is suggestive of a tetramer. While the top panel illustrates the time-dependent evolution of the radius distribution, the bottom panel displays histograms derived from the entire data set. **C** In order to further characterize the oligomerization process of the C-region and the accompanying structural changes, we performed DLS measurements (top panel) along with CD spectroscopy (bottom panel) at various temperatures, 20 °C (black), 30 °C (red), 37 °C (orange), 45 °C (green), 50 °C (blue), and 60 °C (cyan). One additional spectrum (yellow) represents the CD profile of the freshly prepared sample.

From a structural perspective (Fig. 1A), evaluation of the amino acid sequence using bioinformatics analysis indicates that the DISC1 N-terminal region of approximately 350 residues is intrinsically disordered, harboring four distinct phosphodiesterase 4 (PDE4) binding sites [16, 17] and two nuclear-localization signals [18]. The C-terminal part is predicted to be largely helical, including several coiled-coil regions [8, 19] characterized by the presence of heptad repeats (commonly denoted *abcdefg*, with positions *a* and *d* carrying hydrophobic residues and others being either polar or charged). Additionally, this segment has been proposed to contain antiparallel helical hairpin structures known as UVR domains in regions 343–394, 574–625, and potentially also 778–828 [19]. The latter has been confirmed by solution NMR spectroscopy (see below). A systematic search for stably folded and soluble regions using the high-throughput Expression of Soluble Proteins by Random Incremental Truncation (ESPRIT) technique [20] revealed four putative domains that were mnemonically named D, I, S and C and comprise residues 257–383, 539–655, 635–738, and 691–836 [21], respectively. Among these presumably structured regions of DISC1, the C-region has been characterized most thoroughly, including its mode of binding to a camelid nanobody [22]. Solution NMR structures of a truncated version of the murine DISC1 C-region comprising the distal 73 residues have been determined in the presence of peptide fragments of ATF4 [23] and NDE1 [24], respectively. The interaction between the C-region and its binding partner is similar in both systems and involves formation of a three-helix coiled-coil structure, with the helical fragment of ATF4/NDE1 clamped by hydrophobic interactions of the heptad repeats with the two antiparallel helices contributed by DISC1.

From a pathophysiological perspective, the C-region of DISC1 deserves particular attention since it is affected by alterations implicated in mental illnesses. Specifically, this segment is deleted in the Scottish variant [2] lacking residues 599–854 as a consequence of a balanced translocation, and it harbors the sites of a mental illness-associated polymorphism (S704C [25]) and of a frameshift mutation (affecting residues downstream of L807) that results in aggregated complexes [7]. A phosphorylation site observed at S713 [11] serves the role of a switch that promotes the transit of neuronal progenitor cells from proliferation to migration during corticogenesis. Moreover, other disease-associated proteins such as NDE-like 1 (NDEL1) and lissencephaly 1 (LIS1), whose impaired function results in microcephaly and lissencephaly, respectively, interact with these regions [26] alongside the motor protein dynein, all of these being crucial for mitosis. Despite increasing insight into the cellular processes that DISC1 is involved in, its precise molecular functions and their regulation remain poorly understood. Contributing to this is the propensity of the DISC1 protein to aggregate [10], which is the major bottleneck impeding biophysical characterization of the native form and, in particular, determination of its 3D structure. This has led to several unanswered questions: Is it possible to structurally characterize DISC1 aggregates? Why does the protein aggregate and how can this process be prevented? Is the molecular nature of aggregation unique or does it share common features with other proteinopathies? How does aggregation of DISC1 affect its interaction with binding partners and the respective cellular functions?

Herein, we address these queries. To comprehend the impact of DISC1 on the pathophysiology of schizophrenia and related CMIs, we focus our efforts on the C-region of the protein. Employing a combination of biophysical techniques probing structure and dynamics, we demonstrate that the C-region gives rise to cross β-fibril-like aggregates and report kinetic and thermodynamic parameters of their formation. Moreover, we show that the higher oligomeric structures differ from the monomeric species regarding protein–protein interaction properties, suggesting severe consequences for cellular signaling. Altogether, our approach provides us with a biophysical basis to understand the relevance of the C-region of the DISC1 protein with respect to both its physiological role as a scaffold protein and its pathophysiological significance in fibril assembly, in relation to other amyloidogenic diseases.

## Methodology

### Protein expression and purification

The human DISC1 C-region (residues 691–836) was expressed alternatively as N-terminal His_6_- or MBP-His_6_-fusion construct. The His_6_-tag construct was expressed as described previously [22] using the vector pESPRIT002 [20] for transforming *E. coli* BL21 (DE3) pLysE T1R cells, while the MBP-His_6_ fusion construct was cloned in pET-15b and expressed in *E. coli* (DE3) C43 cells. The culture was grown either in Luria-Bertani (LB) broth or M9 minimal medium. Protein expression was induced at an OD_600_ of 0.6 by the addition of 1 mM isopropyl-*β*-d-thiogalactopyranoside (IPTG) for 16 h at 18 ˚C. Harvested cells were lysed in Tris-buffered saline (TBS) buffer containing 10 mM Tris-HCl, pH 7.4, 150 mM NaCl, and Complete EDTA-free protease-inhibitor cocktail (Roche) using an ice-chilled microfluidizer M100P (Microfluidics MPT) at 15,000 PSI. The insoluble fraction was spun down by centrifugation at 50,000 × *g* for 25 min at 4 °C. The soluble fraction was purified on NTA resin (Qiagen) loaded with Co^2+^ and was eluted using TBS containing 500 mM imidazole. In the subsequent step, the His_6_-C-region protein was further purified on a HiLoad 16/60 Superdex 200 size-exclusion chromatography column (GE Healthcare). The MBP fusion protein was purified via an amylose column (New England Biolabs) and eluted using TBS containing 25 mM maltose.

Proteins V_H_H B5 and NDEL1 were prepared in a similar fashion as described previously [22, 27], which was also adopted for LIS1 expression and purification. Briefly, V_H_H B5, LIS1, and NDEL1 proteins were expressed in *E. coli* cells of the strain types Rosetta (DE3) pLysS, C43 (DE3), and BL21 (DE3) pLysE-T1R, respectively. The cells were cultivated at 37 °C until an OD_600_ of 0.6 was reached, following which expression was induced using 1 mM IPTG and maintained for 16 h at 18 ˚C. The harvested cells were lysed as described above, and the proteins were purified on Ni^2+^-NTA resin (Qiagen), eluted using TBS containing 500 mM imidazole, and purified further on a HiLoad 16/60 Superdex 200 SEC column (GE Healthcare).

### Dynamic light scattering (DLS)

Measurements were performed using a SpectroSize 300 (XtalConcepts) instrument and a sample volume of 500 μl. The temperature dependence (20–60 °C) was investigated at a protein concentration of 10 µM, while concentration-dependent aggregation (10–90 µM) was studied at a temperature of 37 °C. Prior to measurements, all samples were centrifuged at 21,000 × *g* for 30 min at 4 °C. Diffusion coefficients were obtained from analysis of the decay of the scattered-intensity autocorrelation function and were used to determine apparent hydrodynamic radii via the Stokes–Einstein equation.

In order to assess the thermodynamics (also see below) of fibrillization, we employed a simple 1D “crystallization” model of amyloid formation [28–30]. The model assumes that upon completion of the process, the solution has converted into a suspension of highly ordered fibrils that are at equilibrium with residual monomers/oligomers. The basic reaction describing the association/dissociation of a single unit to/from an existing fibril can be represented as

1 $$C + F_{i - 1} \rightleftharpoons F_i$$

with equilibrium constant

2 $$K_F = \frac{{\left[ {F_i} \right]}}{{\left[ C \right]\left[ {F_{i - 1}} \right]}} = \frac{{k_{\rm{ass}}}}{{k_{\rm{diss}}}}$$

where *C* represents a single element (monomer or small oligomer) of the DISC1 C-region, *F*_*i−*1_ and *F*_*i*_ stand for fibrils (more precisely, docking sites on fibrils) composed of *i*−1 and *i* units, respectively, and *k*_ass_ and *k*_diss_ are the corresponding rate constants. In the later stages of the reaction, the number of growing fibrils will be largely constant because the depletion of soluble material will limit further nucleation; given that fibrils are unbranched in our case (see below), the number of association/dissociation sites, which are presumably located at the fibril ends, will likewise be constant. In this situation, the concentration of docking sites effectively becomes independent of fibril length *i*, and eq. [2] simplifies to

3 $$K_F = \frac{1}{{\left[ C \right]}}$$

Determination of [*C*], the equilibrium concentration of C-region monomers/oligomers, which is popularly referred to as the “critical concentration”, is not straightforward owing to the extensive heterogeneity of the system. Considering that radii corresponding to these building blocks were fitted to the DLS data of fibril suspensions only intermittently, we reasoned that their concentration should be on the order of the detection limit of the instrument, which was determined experimentally. From the resulting equilibrium constant *K*_*F*_, we can compute the apparent free-energy change of fibrillization Δ*G*_app_ = −*RT* ln *K*_*F*_ = *RT* ln [*C*], where *R* and *T* are the gas constant and temperature, respectively.

### Isothermal titration calorimetry (ITC)

Measurements were performed using an iTC-200 (MicroCal) calorimeter with a 200 µl sample cell. All protein samples were degassed prior to measurements. Changes in the heat flow were monitored in real time with the reaction cell stirred at 300 rpm and reference power of the cell set to 5 μcal·s^−1^.

Thermodynamic parameters were computed on the basis of the Goto–Kardos scheme [29, 30]. Assuming that the observed heat exchange represents the enthalpy change of association of the DISC1 C-region, ΔH at 37 °C was calculated by integrating the respective peak area, followed by normalization against protein amount. The entropy change ΔS was calculated using the values of ΔH and ΔG_app_ using the Gibbs–Helmholtz equation (ΔG_app_ = ΔH – TΔS, assuming ∆H is independent of temperature to a first approximation). Likewise, the heat-capacity change at constant pressure (ΔC_p_) was obtained by linear extrapolation of the values from (δΔH/δT)_p_, based on experiments that were performed for the spontaneous fibrillization of 10 µM protein at temperatures ranging from 20 to 60 °C. The reference cell contained filtered and degassed water.

### Thioflavin-T (ThT) fluorescence assay

The assay was performed on black nonbinding 96-well plates (Sigma-Aldrich) using a total volume of 100 μl containing 10 μM ThT per well, and protein concentrations ranging from 5 to 90 µM. Different temperatures (25 °C, 37 °C, and 45 °C) were evaluated with a protein concentration of 10 µM. Each reaction was background-corrected by subtraction of the buffer control. Fluorescence was monitored at regular intervals of 3–5 min using a Fluostar microplate reader (BMG Labtech, Offenburg, Germany) with 440 nm excitation and 492 nm emission filters, respectively, in bottom-read mode.

### Circular dichroism (CD) spectroscopy

Far-UV CD spectra were recorded on a Jasco J-1100 spectropolarimeter at 20 °C (0.1 cm path-length cuvette) in TBS at a ramp rate of 50 nm min^−1^. Prior to the measurements, protein samples were centrifuged at 20,000 × *g* for 20 min at 4 °C to remove large aggregates and fibrils. Spectra were individually normalized to the ellipticity at wave length of 217 nm.

### Surface plasmon resonance (SPR) spectroscopy

Binding of LIS1 and NDEL1 to the C-region constructs was investigated using a Biacore T200 instrument (GE Healthcare). Prior to measurements, all protein samples were dialyzed against phosphate-buffered saline (PBS) containing 20 mM Na-phosphate, pH 7.4, and 150 mM NaCl. For coupling of the ligand, the flow cells on a CM5 sensor chip (GE Healthcare) were activated with 1-ethyl-3-(3-dimethylaminopropyl) carbodiimide (EDC)/N-hydroxysuccinimide (NHS) (200 mM/50 mM); the DISC1 C-region fragment was diluted to 30 μg/mL in 10 mM sodium acetate, pH 4.0, immobilized to a final level of 250 RU, and the flow cell was deactivated with 1 M ethanolamine-HCl. A reference flow cell was treated in a similar fashion. Titrations of the above-mentioned interaction partners were performed at 25 ˚C with PBS, pH 7.4, as running buffer, using protein concentrations ranging from 0.75 to 10 µM without intermittent regeneration. The sensorgrams were double-referenced using the reference flow cell and a buffer cycle, while evaluation was performed by plotting the respective equilibrium-response level against the applied analyte concentration. The curves were individually fitted using Hill’s equation (OriginPro 2020).

### NMR experiments

Solution NMR experiments were performed at a static magnetic field strength of 17.6 T, corresponding to a ^1^H Larmor frequency of 750 MHz, using a Bruker Avance III spectrometer equipped with a 5 mm cryogenic triple resonance probe. Measurements were conducted at 298 K. The protein sample was provided in PBS buffer (20 mM Na-phosphate, pH 7.4, 150 mM NaCl) containing 10% (v/v) D_2_O for field-frequency locking. 2D ^1^H–^15^N HSQC spectra were recorded to obtain a fingerprint of the protein and assess the highly dynamic protein regions visible to solution NMR. Decoupling of ^15^N during proton acquisition was performed by application of the GARP scheme. Solvent suppression was achieved using the conventional WATERGATE scheme. ^1^H chemical shifts (CS) were referenced directly to sodium trimethylsilylpropanesulfonate (DSS) at 0 ppm and ^15^N shifts were referenced indirectly to DSS, using the absolute-frequency ratios. The HSQC spectra were processed using a squared sine bell function of 2. Additionally, 2D ^1^H–^15^N HSQC was recorded to follow the aggregation of the DISC1 protein in solution (three different concentrations) at various time points. Furthermore, to analyze the concentration dependence, HSQC spectra of proteins were recorded at three different concentrations of 10, 60, and 400 µM, respectively, in a time-dependent manner.

Solid-state NMR (ssNMR) experiments were performed on a uniformly ^15^N^13^C-labeled sample of the DISC1 C-region, dissolved in TBS buffer containing 1 mM Na_2_-EDTA and 0.01% (w/v) NaN_3_, which was concentrated in a centrifugal device with 3 kDa cutoff membrane (Amicon, Millipore). The concentrated sample was bath-sonicated for 5 min and incubated at 20 °C for 2 weeks. At this point, it contained noticeable particulate matter, had a consistency similar to hydrogel, and was packed into 1.9- and 3.2-mm zirconia magic angle spinning (MAS) rotors (Bruker Biospin). Measurements were conducted at static magnetic field strengths of 16.4 and 18.8 T (corresponding to ^1^H Larmor frequencies of 600 and 800 MHz, respectively) and an experimental temperature of 256 K, using 1.9- and 3.2-mm triple-resonance (^1^H,^13^C, and ^15^N) Bruker MAS ssNMR probes (Bruker Biospin). Cross-polarization (CP)-based experiments, sensitive to more rigid regions of the protein, were performed at 600 MHz using the 3.2 mm probe. 2D ^13^C–^13^C proton-driven spin-diffusion (PDSD) experiments were recorded at 12.5 kHz MAS frequency with 20 ms mixing time and under weak coupling conditions [31], with mixing time of 20 and 200 ms at MAS frequencies of 8.5 kHz, respectively. ^1^H decoupling was applied during evolution and detection periods, using the SPINAL64 scheme at a radio-frequency between 66 and 83 kHz.

To obtain information on the highly dynamic regions of the protein, 2D ^1^H–^15^N as well as ^1^H–^13^C correlation experiments, employing refocused insensitive nuclei enhanced by polarization transfer (refocused INEPT)—with detection on the ^15^N and ^13^C nuclei, respectively, were recorded. This was complemented by the proton-detected version of the 2D ^1^H–^15^N as well as ^1^H–^13^C correlation experiments [32], employing refocused INEPT transfers as well. Experiments were conducted at 800 MHz and a MAS frequency of 30 kHz (using the 1.9 mm probe). During acquisition of the ^1^H dimension, a 10-kHz weak-power decoupling was applied on the ^15^N and ^13^C nuclei using the WALTZ64 scheme. During evolution of ^15^N (or ^13^C) nuclei, a 10 kHz weak power proton decoupling was applied using the frequency-swept TPPM scheme. Solvent suppression was achieved by the MISSISSIPPI scheme.

Further, ^13^C–^13^C total through-bond correlation spectroscopy (TOBSY [33]) with 6 ms mixing at 16 kHz MAS was recorded at 800 MHz. Low-power ^1^H decoupling using the sequence TPPM at 10 kHz was applied during the detection period. ^13^C and ^15^N CS were referenced using the most downfield signal of Cα and N in the tripeptide MLF [34], which is 55 and 100 ppm, respectively.

Water-edited 1D build-up experiments were recorded at 600 MHz and 12.5 kHz MAS, the temperature was maintained at 256 K. A ^1^H T_2_ filter of 2.5 ms was used to suppress signals from the rigid regions of the protein; for the ^1^H–^1^H spin diffusion, mixing times (*t*_*m*_) of 2–500 ms were used to permit spin diffusion from water to the protein. The spectral region of 50–75 ppm was integrated and normalized to the maximum signal intensity (at 100 ms mixing time). A linear fit to the initial build-up rate ($t_m^s$) was used to determine the water accessibility of the sample. The slope describes the time required to reach 100% magnetization transfer in the absence of any saturation effects [35] and is inversely proportional to the volume-to-surface area ratio, which is described by $\frac{V}{S} = \sqrt {\frac{{D_{\rm{eff}}t_m^s}}{\pi }}$ where *V* represents the volume of the protein, *S* is the surface area of the protein accessible to water, and *D*_eff_ is an effective magnetization diffusion coefficient corresponding to 0.2 nm^2^/ms [36, 37]. Assuming that the fibril represents an elongated cylinder, where fibrillary length is significantly larger than the diameter (*d*) of the fibril, the ratio of *V/S* equals *d*/4. Such an approach provides a semiquantitative estimate to the molecular dimensions of water accessible areas of membrane proteins [36] and amyloid fibrils [37] in comparison with their low-resolution structural models. To determine amino acid specific hydration, we employed water-edited 2D ^13^C–^13^C experiments with mixing times of 20 ms to encode intraresidue correlations with the conditions mentioned above. Intensities for regions of interest corresponding to CS of residues in different secondary structural elements (SSE) were integrated. The resulting signals were normalized to the number of scans and signal intensity of the particular amino acid in the respective SSE for a comparison between different ^1^H–^1^H mixing time.

All data were processed by Topspin 4.0.6 (Bruker Inc.), CS was predicted using the FANDAS [38] software and analyzed using NMRFAM-SPARKY [39].

### Transmission electron microscopy (TEM) measurements

The sample solution (4 µl) containing 10 µM protein was applied onto a glow-discharged carbon-coated copper grid (S160-4, Plano). After 2 min, the solution on the grid was blotted off by filter paper. Then the grid was washed with 4 µl of 1% (w/v) uranyl acetate (UrAc) and blotted off immediately, and another 4 µl of 1% (w/v) UrAc was applied onto the grid for 1 min. Then the solution was blotted off and the grid air-dried. TEM images were obtained using a TFS Talos 120 C (Thermo Scientific) with a voltage of 120 kV. Processing of the negatively stained images was performed using RELION 3.1.0 [40] and the contrast transfer function (CTF) was fitted using CTFFIND4 [41]. For the tetramer, 40 particles were selected from ten images (pixel size of 2.5 Å) and extracted with a box size of 80 pixels (200 Å). For the fibrils, 14 fibrils were selected from three images (pixel size of 1.55 Å) and extracted with a box size of 130 pix (201.5 Å), resulting in 393 fibril segments. Subsequently, 2D classification of the tetramers and the fibrils was performed with a mask diameter of 180 Å.

### Structural models

The template for the protomer of the DISC1 C-region was obtained from a model published previously, which is consistent with SAXS data [22]. The proposed β-structure was introduced into the model using MODELLER 9.23 [42] after adding restraints defining antiparallel β-strands in segments 716–737 and 739–761, respectively, via the *model‐addrsr.py* script. The newly derived structure was subjected to in silico docking simulations using CLUSPRO, targeting tetramers and oligomers in the multimer docking mode [43]. The resulting models were examined for consistency with the results obtained from ssNMR, EM, and SPR measurements and the best candidate selected for further interpretation.

For comparison of the EM-based 3D reconstructions to the structural models, a 3D-density map was simulated for each model using the corresponding PDB file with EMAN2 [44]. Correlations between the reconstructed 3D-density maps and the simulated-model maps were calculated using UCSF Chimera [45].

## Results

### The DISC1 C-region self-associates into oligomers and aggregates

A DISC1 construct comprising the C-terminal region 691–836 and carrying an N-terminal hexa-histidine tag was studied. While investigating freshly prepared protein using spectroscopic methods, we noticed formation of large particles during the course of the measurements. As a first approach to characterize this particle formation, we performed DLS experiments at different temperatures and protein concentrations. We observed that the protein, which initially (i.e., freshly prepared off-column) displays an apparent hydrodynamic radius (R_H_) in the range of 2.12–3.11 nm (corresponding to a molecular mass (M) of approximately 14–41 kDa for a globular protein), tends to associate into larger particles in the range of 100 nm within a few hours at 20 °C (Fig. S1). Given a theoretical mass of the His_6_-tagged protein of 19.4 kDa, the major species would seem to represent a monomer or a dimer (or both), but this assignment may be confounded by the elongated shape of this fragment [22]. Intriguingly, DLS measurements at 37 °C, using protein concentrations ranging from 10 to 90 µM, revealed a predominant fraction with an apparent R_H_ of 4–4.5 nm (81.3–99.8 kDa), which may correspond to a tetramer, and another stable population at approximately 13–19 nm (1.2–2.8 MDa) that became more apparent with increasing concentration (Fig. 1B). A systematic investigation of temperature dependence of the aggregation profile of the DISC1 C-region (Fig. 1C) showed a smooth transition of the presumed tetramer into several relatively well-defined species with radii above 100 nm, especially at temperatures below 37 °C. At temperatures higher than 37 °C, we do not observe any consistent aggregate sizes but radii scattered across the nm–µm range. The profile at 37 °C, however, was remarkably stable over the measurement duration with tiny-to-negligible scattering fluctuations. In combination with DLS recordings, we performed CD measurements to investigate potential structural transformations at various temperatures (Fig. 1C). Notably, at temperatures lower than 37 °C, we observe a robust change in spectral shape affecting the ratio of the minima at 208 and 222 nm. This transition may reflect loss in helicity and increase in the β-content of the system. In order to ascertain this assumption, we performed ssNMR spectroscopy experiments (*vide infra*) to confirm on the presence of β-strands. At higher temperatures though, the pattern is inconsistent at the characteristic minima of 208 and 222 nm and difficult to rationalize. Perhaps, the spectra represent a mixture of populations with varying secondary-structure element (SSE) composition. Interestingly, alike DLS measurements, the CD spectra at 37 °C agreed well with the freshly prepared sample. In order to ascertain that the tetrameric state represents an intrinsic property of the DISC1 C-region and not an artifact related to the His_6_-tag, we generated an alternative construct containing MBP as fusion partner, which should also render the protein more soluble and may thus (at least to some extent) counteract self-association promoted by nonphysiological protein concentrations. Similar to our observations with the His_6_ construct, the MBP-tagged version of the DISC1 C-region predominantly existed as a tetramer in solution at different temperatures (Fig. S1). Based on these observations, we hypothesized that the tetramer constitutes a physiologically significant oligomeric state, whereas the larger aggregates represent aberrant forms that may relate to pathology.

To further dissect the relevance of the aggregation phenomenon that we observe at temperatures lower than 37 °C, we proceeded to identify the critical concentration of the DISC1 C-region assuming the 1D crystallization model of amyloid formation [28–30]. The inkling here is that the concentration of fibrils remains stable as the oligomers and monomers are constantly consumed during elongation. As a result, the equilibrium constant (*K*_*F*_) for fibril formation can be approximated by the inverse of the concentration of protein in equilibrium with the fibril suspension, i.e., the critical concentration (also see “Methodology” section pertaining to DLS measurements), which was observed as 750 nM (Fig. S1). Using this value for the critical concentration, we estimated the ΔG_app_ of fibril formation as −34.36 kJ mol^−^^1^. We proceeded to perform a thorough biophysical characterization of the different oligomeric states observable via DLS, in order to obtain insight into their architecture and functional properties.

### Architecture of the C-region of DISC1

**Fig. 2:**
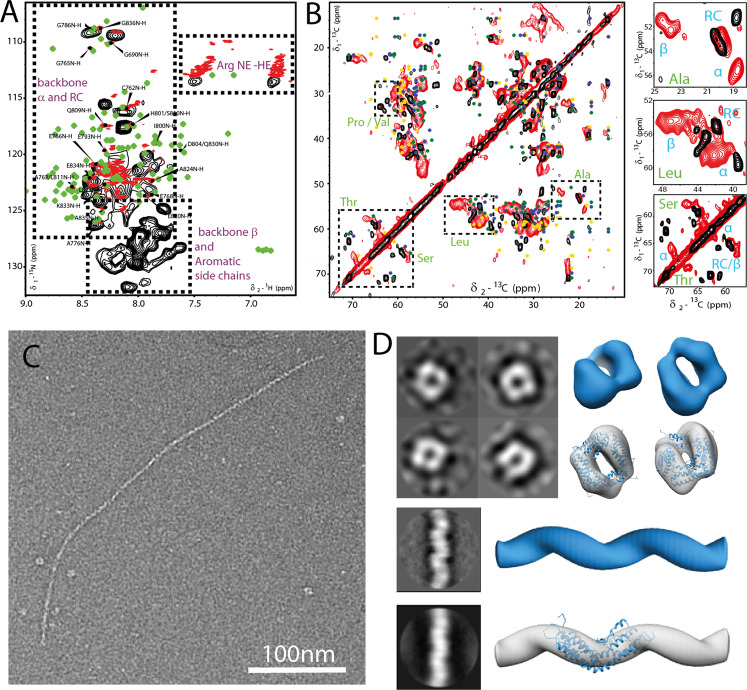
Structural characterization of the DISC1 C-region using a combination of NMR spectroscopy and EM. In all spectra displayed in the figures, the protein was uniformly ^13^C- and ^15^N-labeled. **A** 2D (^1^H–^15^N)-HSQC spectra of the C-region in solution (red) and solid state (black) at a MAS rate of 30 kHz. Green diamonds represent solution NMR CS of the truncated version of the DISC1 C-region (PDB ID 5YIH) and black annotations are likely assignments of C-region resonances based on proximity to the solution NMR signals. **B** 2D PDSD (red) and INEPT-TOBSY (black) ^13^C–^13^C spectra, diamond-shaped marks in yellow, blue, and green represent average CS (ref. [47]) of residues in helices, coiled regions (RC), and strands, respectively. The box depicts the region where the presence of β-strand-specific CS is expected in dipolar coupling-based experiments for residues such as Ala, Leu, and Ser. **C** Negatively stained EM image of the DISC1 C-region showing the presence of fibrillar protein along with oligomers. **D** 2D class averages of the tetramer and the fibril. For better visualization of the overall fibrillar structure, one class (top) was calculated by ignoring the CTF until the first peak. The 3D reconstructions of the tetramer and the fibril are displayed as surface representations alongside the best-fitting structural model docked within the density map (also see Figs. 4 and S8).

As mentioned above, bioinformatics [8, 19] analysis highlights the predominance of coiled-coil arrangement in the DISC1 C-region, which is experimentally supported by structural information [23, 24] obtained on a truncated version of the murine homolog (73 residues ranging from 764-836); however, the entire C-region comprising 160 amino acid residues has been intractable for structural characterization. In our first attempts using 2D NMR spectroscopy (^1^H–^15^N HSQC), we noticed that well-defined resonances were grossly under-represented. Signal dispersion is poor and the characteristics of folded protein are not visible. Interestingly, for the few resolved peaks (Fig. 2A), the observed chemical shifts (CS) agree well with the solution NMR data published previously [24] on the truncated murine version mentioned above, and largely localize to the segment spanning residues 790–830 (Fig. S2). More importantly, the broadened signals may indicate oligomerization or the presence of disordered regions or a combination thereof. Regardless of the protein concentration (Fig. S2), we noticed broadened resonances in all tested conditions and for the few resolved peaks, we observed CS perturbation in a time-dependent manner. Altogether, these findings are suggesting a tendency of the protein to aggrandize, consistent with our observations from the DLS measurements mentioned above. Hence, we believe that the solution NMR data reflect the presence of tetramer that is steadily fibrillizing.

At the same time, we performed ssNMR spectroscopy measurements to separately probe dipolar transfer and scalar couplings (Fig. 2B) on the gel-like sample that is predominantly fibrillar in equilibrium with the tetramer. We observed an overwhelming signal in the INEPT transfer in comparison with the CP-based spectrum, indicating that the C-region comprises elements that are highly mobile on the ssNMR timescale. A close inspection of dipolar-based 2D ^13^C–^13^C transfer data (Fig. 2B), which encode intraresidue correlations from rigid parts of the protein, highlights select residues, notably Ala, Gly, Leu, Pro, Ser, Thr, and Val, with CS reminiscent of residues in β-strands. On the other hand, 2D spectra using ^13^C–^13^C-INEPT-TOBSY (Fig. 2B) and ^1^H^13^C-INEPT (Fig. S3) correlations exhibit signals exclusively due to amino acids in helices and coils, and are completely devoid of resonances arising from residues in β-strands. To complement the INEPT-transfer experiments, we performed ^1^H detected ssNMR experiments [32, 46] alike solution NMR ^1^H^15^N- and ^1^H^13^C-HSQC but at medium MAS rates of 30 kHz. We observed a high degree of overlap (Fig. 2A) between the solution and solid-state ^1^H–^15^N correlations, strongly supporting the notion that the flexible component of the C-region is located in the region comprising residues 764–836. The ^1^H–^15^N correlations at 30-kHz MAS also displayed back-folded signals due to lysine side chains. These sets of experiments are well poised to probe various ranges of protein dynamics that are relevant for protein function and structure (*vide infra* for water-edited spectra). Also, similar to the ^13^C–^13^C correlation spectrum, the ssNMR-HSQC experiments (Fig. S3) are dominated by signals arising from residues in helices and coils, while a CP-based ^1^H–^15^N transfer (Fig. S3) at 30-kHz MAS, albeit inhomogeneously broadened, also shows the presence of CS that is predicted for residues in β-strands [47].

To investigate the molecular architecture of the aggregates, we complemented NMR experiments with EM studies. The monomeric DISC1 C-region has a calculated molecular mass of 19.4 kDa, which roughly corresponds to a hydrodynamic radius of 2.28 nm. In negative-contrast electron micrographs, however, we observed two major populations representing higher oligomers and fibrils, respectively (Fig. 2C), and also noted the presence of amorphous aggregates (Fig. S4). Most of the oligomeric particles in the preparations displayed apparent cyclic symmetry, the predominant species being C4 with an average diameter of 10–12 nm, while minor populations suggesting C5 and C6 were also observed. The 3D reconstruction of the tetramer (Fig. 2D, upper left) is suggestive of few contacts between the subunits and a pore in the center. Fibrils on the other hand appeared as either short segments measuring 100-200 nm in length or longer filaments that extended up to tens of micrometers. The density map of the fibril (Fig. 2D, lower left) is indicative of a dimeric repeat across the length of the structure. The fibrils generally displayed a helical twist with a pitch and average diameter of approximately 9–12 and 5–7 nm, respectively.

### Thermodynamics of fibrillization

**Fig. 3:**
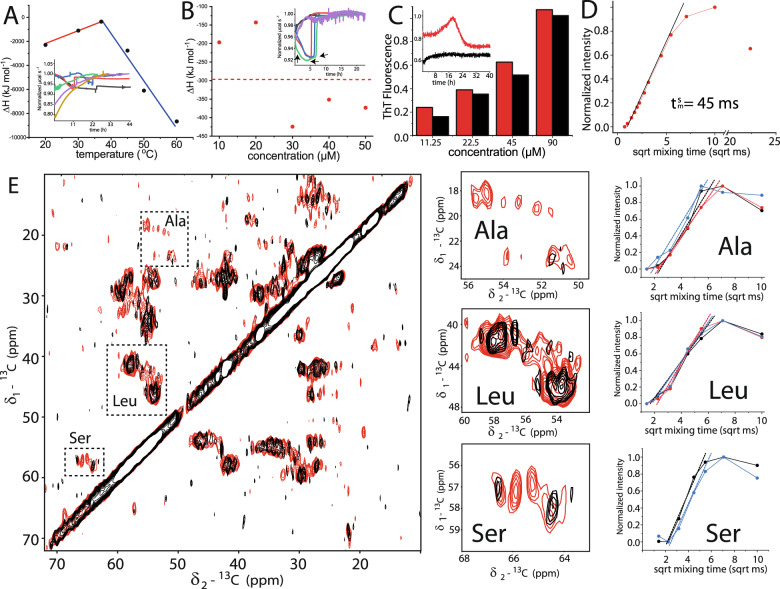
Mechanistic and thermodynamic characterisation of C-region aggregation. **A** Thermodynamic characterization of the DISC1 C-region via ITC. The enthalpy changes observed for a 10 µM protein solution are plotted as a function of temperature; the inset shows the corresponding thermograms (20 °C, black; 30 °C, red; 37 °C, blue; 45 °C, green; 50 °C, purple; and 60 °C, golden). Heat capacities (ΔCp) of 113.84 (red) and −373.51 (blue) kJ mol^−1^ K^−^^1^ were derived from the slopes of the two phases using a linear fit. **B** The enthalpy change (ΔH = −297.74 kJ mol^−1^) was calculated by normalizing the observed heat changes in the thermograms by protein amount (in inset; 10 µM (black), 20 µM (purple), 30 µM (red), 40 µM (blue), and 50 µM (green) protein solutions). **C** ThT assay highlighting the fluorescence intensity (in arbitrary units) of the DISC1 C-region at 25 °C (red) and 37 °C (black) as a function of protein concentration. The inset figure shows the raw data obtained at 45 µM protein concentration (also see Fig. S5). **D** 1D water build-up curves of CP intensity in predominantly Cα region corresponding to signals in the region 50–75 ppm (Fig. S6). Dotted line represents the fitted slope that was used for estimation of $t_m^s$. **E** Left panel displays 2D ^13^C–^13^C correlation spectra with ^1^H–^1^H mixing times of 10 ms (black) and 100 ms (red), the right panels depict close-up views for three spectral regions corresponding to the signals from residues Ala, Leu and Ser and their respective water build-up profiles at different ^1^H–^1^H mixing times (refer to Fig. S6 for individual spectrum). Corresponding signals arising from secondary-structure elements (SSE) like helices, strands, and coils are color-coded in red, blue, and black, respectively. The corresponding colored dotted lines represent the slopes arising due to individual SSE and were used further for estimation of $t_m^s$.

In ITC measurements (Fig. 3A, B), at concentrations of 10 µM and at 37 °C, we observed a weak-intensity exothermic burst, and ThT-fluorescence studies revealed a steady rise in the signal with a plateau that was reached after 8 h (inset of Fig. 3C, black trace). Under this condition, we had previously detected few long fibrils as well as oligomers in electron micrographs (Fig. S4), which is also supported by DLS experiments. At 25 ^o^C, we observed an increase in the ThT fluorescence signal over a period of 8–40 h (Fig. S5) and a subsequent gradual decline below the baseline (inset of Fig. 3C, red trace). Indeed, EM studies highlight that the length of fibrils is temperature-dependent and the ones grown at lower temperatures (such as 20 °C) are considerably longer than those cultivated at higher temperatures. We hypothesize that the time-dependent reduction in fluorescence signal accompanying the formation of longer fibrils may arise due to increased precipitation and/or adhesion to the plate walls, compared with conditions where numerous smaller fibrils are formed. At temperatures above 37 °C, in all conditions tested using ITC, we observed a pronounced exothermic reaction. Alongside, the ThT assay at 45 °C revealed a slow tonic increase in fluorescence like the one observed at 37 °C, but with far weaker intensity (Fig. S5); this observation is in agreement with both EM and CD measurements revealing amorphous aggregates and formation of several short fibrils respectively, at this temperature (Figs. 1C and S4). Furthermore, in DLS experiments conducted at 60 °C we did not observe a distinct population but larger particles in nm–µm dimensions, while electron micrographs revealed (Fig. S4) the presence of bundles and clusters of short fibrils. Taken together, this would imply that at higher temperature, the DISC1 C-region undergoes nucleation at an enhanced rate, resulting in fibrils that are more numerous, heterogeneous, and shorter in length because of a rapidly decreasing concentration of free protein, while at lower temperatures, one observes a slow steady nucleation and longer homogeneous fibrillar growth.

Next, we investigated the effect of protein concentration on the heat exchanges during fibrillization at 37 ^o^C. Higher concentrations resulted in isotherms with a broad exothermic burst similar to those for higher temperatures (Fig. 3B). We calculated an enthalpy exchange of −297.74 kJ mol^−^^1^ (Fig. 3B and Table 1**)** at 37 °C, while values ranging from −1.5 to −150 kJ mol^−1^ have been observed for other amyloid systems [29, 30, 48, 49]. Next, we calculated the heat-capacity change for the formation of fibrils and observed steep ΔCp (Fig. 3A**)** trends of 113.84 and −373.51 kJ mol^−1^ K^−1^ (Table 1). In almost all amyloids [29, 30, 48, 49] investigated, one observes a negative tendency of ΔCp during fibrillization; besides tight packing of residues in newly formed β-core regions, this effect may arise from solvent-related phenomena such as desolvation of hydrophobic surface areas and trapping of water molecules at protein–protein interfaces.

**Table 1:** Thermodynamic properties of DISC1 C-region association.

ΔH [kJ/mol]	ΔG_app_ [kJ/mol]	ΔS [kJ/mol/K]	ΔCp [kJ/mol/K]
−297.74	−34.36	−0.9	113.84 & −373.51

Another notable feature in all cases of cross-β-fibrillar proteins is a negative entropy change^24,25,51^, consistent with assembly of highly ordered structures with decreased bulk-solvent access and presence of ordered water molecules. Our calculations of ΔS indeed yield negative values of −0.9 kJ mol^−1^ K^−1^ (Table 1). Therefore, in order to probe the presence of water accessible at the surface of the fibril, we performed water-edited ssNMR experiments (Fig. 3D, E). Previous studies on Aβ [50] have shown that ^1^H T_2_ relaxation times of approximately 250, 150 and 90 ms can describe the presence of water as bulk, interfibrillar, and fiber bound, respectively. Other reports on classical amyloids have shown from build-up experiments that ordered water molecules associated with the core region of β-fibrils [37, 50, 51] in the range of 17–110 ms. Altogether, the interaction between water and the core region occurs in the tens of ms range. While analyzing region 50–75 ppm that is predominated by signals arising from backbone Cα in the 1D water build-up experiment (Fig. S6), we observed that the DISC1 C-region contains tightly bound water and requires initial polarization transfer times $t_m^s$ of 45 ms (Fig. 3D). This would represent the presence of water in close proximity to the surface area. In turn, this would correspond to a fibrillary core with a diameter of 6.8 nm, agreeing well with our observations from the EM images of the fibril (approximately 5–7 nm). We next performed 2D water edited ^13^C–^13^C spin diffusion experiments at various ^1^H–^1^H mixing times ranging from 5 to 100 ms (Fig. S6). A close analysis in the amino acid-specific regions, viz., Ala, Leu, and Ser (Fig. 3E) reveals the following: for the residue Leu, in all tested conditions, we noticed an approximate equidistribution of population intensities for all SSE with an $t_m^s$ of 19 ms for sheets and coils, and 13 ms for helices. In the case of Ser, we noticed signals arising from β-strands and coils that display build-up times of 15 and 11 ms, respectively. Only in the short mixing time of 5 ms, we observe a weak signal for Ser in α-helix (Fig. S6**)**. The steeper build-up profile for Ser is unsurprising considering that the amino acid side chain is polar and favors interaction with water. Resonances from Ala, however, are weakly observed at lower mixing times and increase starkly from 20 ms onward. The steep increase in the signal profile for all three SSE corresponds to build-up times of 13.5–14 ms.

Altogether, our findings highlight that there is no preferential transfer of magnetization from water to the different SSE and residues of the protein. Due to the unavailability of a high-resolution 3D structure of the DISC1 C-region β-core, we are rather limited with the interpretation of amino acid-specific interaction with water. We can, however, readily deduce: first, the water-edited signals arise due to the fibrils and few residues within, which illustrates that the DISC1 C-region exhibits the presence of ordered water molecules. Second, the association of DISC1 C-region into oligomers/fibrils is enthalpy driven alike other amyloid systems [29, 30, 48, 49].

### Oligomerization/fibrillization increases affinity and promotes cooperativity of C-region binding to other physiological partners

The interaction of LIS1 and NDEL1 with DISC1 was studied using SPR, where the C-region protein was immobilized to the sensor chip. We chose to study three different preparations of the DISC1 C-region, in order to assess the impact of oligomerization. As the His_6_-tagged version of the protein readily aggregated, we used a freshly prepared sample of MBP-fused C-region to represent the monomer. In fact, ITC and DLS measurements (Fig. S1) showed that the MBP-C region is more stable than the His_6_-C region, yet with time displays similar tendencies to oligomerize/fibrillize. Samples of His-C-region and MBP-C region incubated at 4 °C for two weeks and two months, respectively, served as representatives of the aggrandized fraction.

**Fig. 4:**
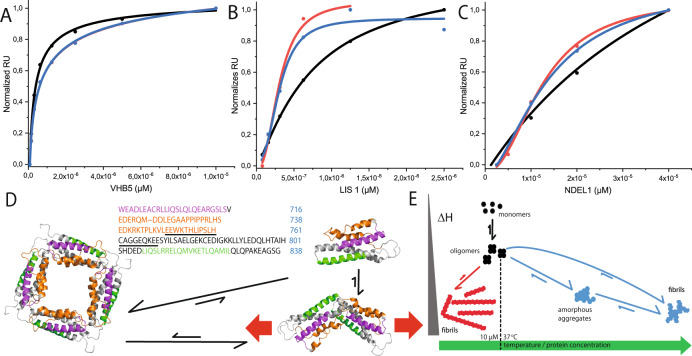
Impact of C-region self-association on physiological DISC1 interactions. Binding isotherms measured using SPR (Fig. S7 and Table 2) representing protein–protein interaction of (**A**) V_H_H B5, (**B**) LIS1, and (**C**) NDEL1 with monomeric MBP-C-region (black), oligomeric/fibrillar His_6_-C region (red), and oligomeric/fibrillar MBP-C region (blue). **D** Representation of structural changes in the DISC1 C-region transitioning from monomer to oligomers/fibrils. Protein sequence is numbered in blue in the context of the full-length DISC1 protein; V_H_H B5-binding site is displayed in magenta, (pseudo)repeat sequence in orange, the underlined part describes residues in Δ22 and the green region represents the C-terminal NDEL1-binding site. The same color coding is used in ribbon representations of protein models and the red arrows indicate directions of filament extension (also see Figs. 2D and S8). **E** Schematic representation of the energy and conformational landscape of DISC1 C‑region self-association. The monomer and/or oligomer versions of the DISC1 C-region show a propensity to fibrillize into longer filaments at temperatures of 37 °C. With increasing temperature and protein concentration, accelerated nucleation yields more numerous but shorter fibrils along with amorphous aggregates. The fibrillar pathways are color-coded according to the temperature domains depicted in Fig. 3 A.

At first, to see if there were any qualitative differences between the monomeric and oligomeric/fibrillary species, we studied binding of a camelid nanobody (V_H_H B5), which we have extensively characterized previously [22] (Figs. 4A and S7). The isotherm was well described using a simple hyperbolic binding model in all three cases, however, both oligomeric fractions showed slightly altered affinities in comparison with the monomeric MBP-C-region. The K_D_ values (Table 2) calculated were 46, 66, and 70 nM, respectively. Similar to our observations with V_H_H B5, the monomeric C-region displayed hyberbolic binding isotherms with K_D_ values of 71 nM and 28 µM to LIS1 and NDEL1 (Fig. 4B, C), respectively. It is important to note, however, that the higher-order oligomers displayed a significant sigmoidal trend, indicating cooperative binding. In the case of LIS1, we determined Hill coefficients of 2.4 and 2.8 along with K_D_ values of 31 nM for both the His and MBP versions, respectively. In a similar manner, NDEL1 displayed cooperative binding (Hill coefficients of 2.1-2.5) together with higher apparent affinities (K_D_ of 13 and 14 µM) for the oligomeric versions. These observations indicate that oligomerization and/or fibrillization of the DISC1 C-region not necessarily lead to sequestration of inactive material, but—at least for certain partners—tend to enhance protein–protein interactions. Potential implications of this behavior are discussed below.

## Discussion

The seminal issue addressed by the current work concerns the question if and to what extent it is possible to structurally characterize DISC1 aggregates. Indeed, our findings reported herein shed light onto several structural and physiological tenets regarding the human DISC1 protein. Using a combination of ssNMR spectroscopy and EM measurements, we are able to show that the C-terminal segment forms oligomers and fibrils, the latter displaying a regular pitch along their axis as well as characteristics of amyloid structure. This piqued our curiosity whether the C-region of the DISC1 protein (perhaps also full-length human DISC1) may behave in ways comparable to other proteinopathies. In fact, one report has described ThT-positive [52] full-length recombinant human DISC1 fibrils, suggesting a potential amyloidogenic nature of DISC1, even though, to this end, DISC1 amyloid has not been reported either in human post-mortem brains or in animal models [12]. Moreover, for nearly a decade, it was believed that DISC1 may resemble other protein risk factors in various neurological disorders [9], however, structural data at atomistic resolution have been severely lacking.

Amyloid systems, including PrP [53], Tau [54], and RIPK1-K3 [55] heteromers to name a few, have a small fraction of their sequences embedded in the β-core, while the remaining larger parts of the proteins often comprise dynamic regions. One exception may be the in vitro-obtained Aβ amyloid-fibril structure [56], where all 42 residues are part of the fibril core. Our findings from solution NMR and INEPT-based ssNMR experiments highlight a dynamic and highly mobile C-terminal end of the protein. On another note, a protomer–fibril equilibrium [57] has been shown to exist in α-synuclein where monomer-dominated signals were observed in INEPT and fibril-derived resonances populated the CP-based spectra. Likewise, it appears conceivable that our observations from the NMR-spectroscopy data may not only represent differential mobility within fibers, but also point to a dynamic equilibrium between oligomers and fibrils, which is indeed well supported by our findings from DLS, ITC, and EM recordings.

This raises several important questions: why does the protein aggregate and which regions of the DISC1 protein contribute to fibrillization? While the ultimate proof is still lacking, our observations together with published data do allow several important conclusions. First, notable signature motifs of amyloid fibrillization require the presence of hydrophobic residues [58] within short peptide sequences [59], repetitions (single amino acid repeats [37] like polyQ; tandem repeats [60]; prion-like repeats [60, 61]; and pseudorepeats [54, 62]) with an intrinsic propensity to form β-strands. While a few of these features have been incorporated in amyloid prediction softwares, these programs were all unsuccessful in determining the β-core interface of DISC1. A manual search, however, for (pseudo)repeats using the program RADAR [63] identified one such long tandem motif covering residues 717–737 and 739–761. This region is abundantly rich in amino acids like Ala, Gly, Leu, Pro, and Ser, which we show have CS reminiscent of residues in strands. Also, the sequence contains hydrophobic residues that may support formation of a β-core.

**Table 2:** Summary of binding constants.

	V_H_H B5 K_D_ [nM] (n)	NDEL1 K_D_ [µM] (n)	LIS1 K_D_ [nM] (n)
Monomer (M)	46 (1.4)	20 (1.4)	71 (1.2)
Oligomer/Fibril (H)	66 (1.1)	13 (2.1)	31 (2.4)
Oligomer/Fibril (M)	70 (1.1)	14 (2.5)	31 (2.8)

Legend: n – cooperativity index, M – MBP fusion construct, H – His_6_ fusion construct.

Next, we sought to address the physiological consequences of aggregation as well as the question whether the oligomer is crucial for DISC1 functioning or represents a pathological species. From a physiological perspective, the pseudorepeat is located in the vicinity of crucial residues such as S704 [25] and S713 [11], on one side, it comprises the Δ22 region [64, 65] (see below) and is flanked by the NDEL1-binding region on the other side. The absence of residues 748-769 as a result of alternative splicing leads to a variant that is referred to as DISC1^Δ22aa^ and displays a marked decrease in binding to NDEL1 [65]. Similarly, it has been convincingly shown by physiological [65, 66] and structural [24] means that region 807–828 is necessary for NDEL1 binding. We speculate that the plasticity surrounding the Δ22/(pseudo)repeat region supports the formation of a β-structure from coil, which may be crucial for different types of self-association, including oligomerization and fibrillization. Notably, our structural models suggest that the resulting conformational change leaves both the N- and the C-terminal portions intact, thus explaining the marginal alteration in the affinity of the nanobody V_H_H B5 [22], which binds in the region 691–715 in a noncooperative manner. Presumptively, oligomerization promoted due to the (pseudo)repeat region may facilitate cooperative binding of physiological partners wherein the binding partner interacts with 2 protomers of the C-region. In a pathological scenario, the same segment could possibly promote fibrillization (Figs. 4D and S8), resulting in a loss-of-function phenotype.

Our findings settle a long-debated question, i.e., whether NDEL1 interacts with monomeric or oligomeric DISC1. Previously, it has been shown that monomeric DISC1 and the S704C-associated oligomers bind to NDEL1 with similar affinities [67]. However, other findings indicated that higher oligomers [10, 68], presumably octamers based on gel-filtration profiles, displayed a greater affinity for NDEL1. We clearly show that, in fact, both results are valid: NDEL1 does indeed bind to both the monomer and the oligomers, but with varying affinities and degrees of cooperativity. That said, it is important to note that the sample representing oligomers may/will also contain fibrils. Does NDEL1 equally bind oligomers and fibers? Perhaps yes, this would indeed be supportive of the loss-of-function phenotype affecting neurite formation [69, 70] and neuronal development [10, 68], which has been observed in conjunction with the precipitation of DISC1. On the other hand, if binding is exclusively associated with the oligomers [10, 68], then they may represent either the physiologically relevant state as present in the dynein complex or a pathogenic species analogous to other amyloid systems, where the fibrils and the aggresomes thereof occur as a nontoxic end product.

Notably, our attempts to study the DISC1 C-region interaction with NDE1 were not straightforward, perhaps due to the propensity of the latter to aggregate into fibrils [27]. It will be interesting to see if the fibrils from the NUDEL system, viz., NDE1 and NDEL1, also contain elements of β-strands. This raises the very pertinent question whether, in addition to the similarities in structural architecture to amyloid diseases, DISC1 aggregation bears any semblance to other known proteinopathies. Akin to aggresomes observed for Alzheimer’s and Parkinson’s diseases, where a multitude of hallmark fibrillar proteins co-aggregate, DISC1opathies [9], i.e., proteinopathies with aggregation or misassembly of the DISC1 protein, may feature a similar tendency for DISC1 and some of its partner proteins. Indeed, our findings on the DISC1 C-region showed the presence of oligomers and fibrils using EM and DLS; biophysical characterization revealed broad enthalpy changes and a negative entropy value using ITC, inhomogeneous line broadening, and mobile and rigid portions of the protein using ssNMR spectroscopy, altogether pointing to heterogeneity and plasticity associated with the protein (Fig. 4E). Interestingly though, amyloid fibrils come in various shapes and architectures. Recently, with the advance in state-of-the-art structural biology techniques and equipment, several new fibril structure details have been reported at atomic and near-atomic level (for review see ref. [71]). It is quite bewildering to note the differences in handedness, fibrillar length, helical axis position, and pitch length. Amyloid fibrils can have widths ranging from 5 to 30 nm and in some cases even hundreds of nm, can be twisted with helical pitches that are extremely short and few may also appear as flat ribbons, with diverse physiological roles (for excellent reviews, readers are referred to [72, 73]). Along these lines, DISC1 C-region fibrils fit well into the grand picture. As far as the driving concentrations for nucleation and aggregation are concerned, in most cases, monomeric concentrations of the amyloid fibril proteins are in the nM to low µM range under physiological conditions, and are tightly regulated by protein synthesis and degradation. Similarly, expression of the DISC1 protein is controlled by the cellular homeostasis machinery. However, under pathological circumstances, an increase of the monomer concentration is likely to occur. This in particular is very interesting because, unlike other amyloid systems where the fibrillary deposits are extensive and readily visualizable by staining techniques, sarkosyl-resistant DISC1 aggresomes obtained from post-mortem brain tissues are highly heterogeneous and usually require enrichment [9, 10]. Contributing to this is the postnatal decrease of expression levels of DISC1 protein [74]. On a side note, due to the poor solubility of the full-length protein, which rendered the system untenable for biophysical characterization, the ESPRIT technique was successfully employed to identify the four more soluble regions of the DISC1 protein [21]. Therefore, based on all the earlier findings and in comparison to the classical amyloids from neurodegenerative diseases, we estimate that the DISC1 monomer may be present in pM to nM concentrations within the cell.

Are the oligomers a functional entity and/or do they perhaps act as seeds for fibril formation? This is an unresolved question, but being a scaffold protein, DISC1 has been shown to influence the activity of a variety of enzymes and other proteins [75]. For myriads of physiological roles, a coiled-coil structure is a very convenient feature offering plenty of conformational flexibility. In the context of our work, DISC1, LIS1, and NDEL1 form a functional triad as part of the dynein complex [26]. It is believed that these proteins mediate opposing interactions wherein LIS1 locks dynein movement, while NDEL1 binding releases it, providing the primary stroke. For these interactions to occur with dynein, a dimeric or tetrameric assembly of LIS1 and NDEL1 is a prerequisite [26]. Also, it has been shown that DISC1 monomers bind NDEL1 [67], likewise, we observe a similar interaction in a noncooperative manner, but that may not be sufficient to promote dynein activity. The C-region of DISC1 has been previously shown to harbor a “multimerization/octamerization” and a “dimerization” domain in regions 668–747 and 765–854 [10, 68]. It has been suggested that oligomerization, presumably octamerization, preferentially promotes NDEL1 binding. This region inherently has a tendency to form sarkosyl-resistant precipitates similar to the ones from brain [10], and the propensity to aggregate is further enhanced by the S704C mutant [68], suggesting a possible disruption of the quaternary structure that may lead to a loss-of-function phenotype. Our findings point in the direction that the structural changes in the region comprising the (pseudo)repeat and the Δ22 (748–769) region likely promote (homo)oligomerization (Fig. 4D). This, in turn, may culminate into cooperative association of DISC1 C-region with NDEL1 and/or LIS1 in promoting dynein activity during the mitotic cycle. However, at this juncture, we cautiously tread on the interplay between the different binding partners because we cannot rule out the possibility of the presence of oligomeric NDEL1 or LIS1 in the analyte solution. It would be very interesting to further dissect if cooperativity arises with monomeric LIS1/NDEL1 or involves (homo)oligomers interacting with the DISC1 C-region (homo)oligomers.

Given that structure and function are two sides of the same coin, our findings help to reconcile divergent results obtained across various experiments and platforms, including inconsistencies with respect to the binding of DISC1 and NDEL1, the mode of interaction with different oligomeric species to name a few. Moreover, the C-region controls proper DISC1 self-association; truncated versions lacking the C-region [1, 2] resemble the disease-associated variant that was observed in the Scottish family with severe chronic mental illnesses. The truncated DISC1 protein associates with the full-length human DISC1, dissociating it from the dynein complex and redistributing it [69], thereby hampering neuronal development [70]. In light of the above, we believe that the results of this study provide a strong foundation for understanding the (patho)physiology of the DISC1 protein in quantitative biophysical terms. Is it possible to prevent the aggregation of DISC1 protein or reverse the process? While our research findings here provide some insight into the potential pathological mechanism, it is too early to answer this question. As one can envision, the findings presented herein have raised a lot more queries than have been addressed. For instance, how can we translate the structural and biophysical information to other mutants of the DISC1 C-region relevant in schizophrenia pathophysiology, such as the S704C, S713E, and L807 (frameshift) to name a few? From the strategy presented herein, the S704C and frameshift mutants represent genetic variants that can be readily targeted for structural characterization, screening for therapeutic applications due to their propensity to misassemble and aggregate. As highlighted above, the S704C mutant has provided a wealth of information from numerous reports on aggregation propensity of DISC1 and its oligomeric specific association with NDEL1 [67, 68]. From the clinical perspective, this mutation has been identified with increased presence of aggregates in brain tissues and is known to be associated with major depressive disorder (MDD), schizophrenia, increase in brain volume, and decreased cognitive function with age [9]. Analogously, another variant that is of clinical significance that was identified due to familial schizophrenia is the frameshift mutant at position L807, which was first identified in the American family [7]. This mutation results in the replacement of amino acids 809–854 with nine residues at the C terminus, resulting in severe aggregation as well as loss of the binding site for the NDEL1 protein. For both the S704C and the L807-frameshift mutants, we speculate that these residues are critical elements of the heptad repeats within the coiled coil region under native conditions. This conserved motif is populated by charged and polar residues such as Ser, and hydrophobic residues such as Leu in positions *a* and *d*; any mutation that destabilizes this structural core may alter the architecture of the Δ22 and adjacent regions, thereby impacting on its binding to other protein partners and eventually resulting in aggregation. Interestingly and in similar vein, other mutants within the Δ22 region such as T754S and P758R result in loss of binding to several key neuronal modulators such as DIX domain-containing 1 (Dixdc1), LIS1, and pericentriolar material 1 (PCM1) to name a few [8]. But given the fact that DISC1 is a scaffold protein, which interacts with over 300 different proteins, the coiled-coil region provides a facile conformational flexibility, especially for post-translational modification as a convenient switch to specify interaction partners. For example, phosphorylation of one crucial site (S713) by protein kinase A (PKA) results in recruitment of Bardet–Biedl syndrome (BBS) proteins to the centrosome, while the dephosphorylated protein interacts with GSK3β in the Wnt signaling pathway [11]. Under such conditions, it is plausible that phosphorylation might disrupt the coiled-coil interactions and facilitate the formation of disordered or β-strand conformations to promote interactions with the BBS proteins; this hypothesis will be subject of our future efforts using biophysical approaches to further comprehend the role of the C-region in DISC1 function. It is important to study the individual regions, i.e., the N-terminal disordered region and the four structured regions, which harbor several sites for post-translational modifications and well-characterized mutations promoting misassembly and aggregation, in a reductionist manner to comprehend their significance in the full-length protein. That said, due to the physiological and psychiatric relevance of DISC1, we are of the firm gestalt opinion that the whole is indeed larger than the sum of individual parts; atomistic detail characterization of the full-length DISC1 protein poses a significant challenge that we intend to tackle in the near future.

## Supplementary information

supplemantary material
