# Supplementary material for: Conformational heterogeneity coupled with β-fibril formation of a scaffold protein involved in chronic mental illnesses
Source: Transl Psychiatry. 2021 Dec 17;11:639. doi: 10.1038/s41398-021-01765-1 (PMC8683410; doi:10.1038/s41398-021-01765-1)
Supplement: Supplementary file 1 — supplemantary material [file 41398_2021_1765_MOESM1_ESM.docx]

**SUPPLEMENTARY INFORMATION**

**Conformational heterogeneity coupled with β‑fibril formation of a scaffold protein involved in chronic mental illnesses**

Abhishek Cukkemane^1,3^*, Nina Becker^1,2,3^, Mara Zielinski^1^, Benedikt Frieg^1^, Nils-Alexander Lakomek^1,2,3^, Henrike Heise^1,2,3^, Gunnar F. Schröder^1,2,4^, Dieter Willbold^1,2,3^* and Oliver H. Weiergräber^1,2^*

^1^Institute of Biological Information Processing (IBI-7: Structural Biochemistry), Forschungszentrum Jülich, Jülich, Germany.

^2^Jülich Centre for Structural Biology (JuStruct), Forschungszentrum Jülich, Jülich, Germany.

^3^Institut für Physikalische Biologie, Heinrich Heine University Düsseldorf, Düsseldorf, Germany.

^4^Physics Department, Heinrich Heine University Düsseldorf, Düsseldorf, Germany

* Corresponding authors – Abhishek Cukkemane ([a.cukkemane@fz-juelich.de](mailto:a.cukkemane@fz-juelich.de)); Dieter Willbold ([d.willbold@fz-juelich.de](mailto:d.willbold@fz-juelich.de)); Oliver H. Weiergräber ([o.h.weiergraeber@fz-juelich.de](mailto:o.h.weiergraeber@fz-juelich.de))


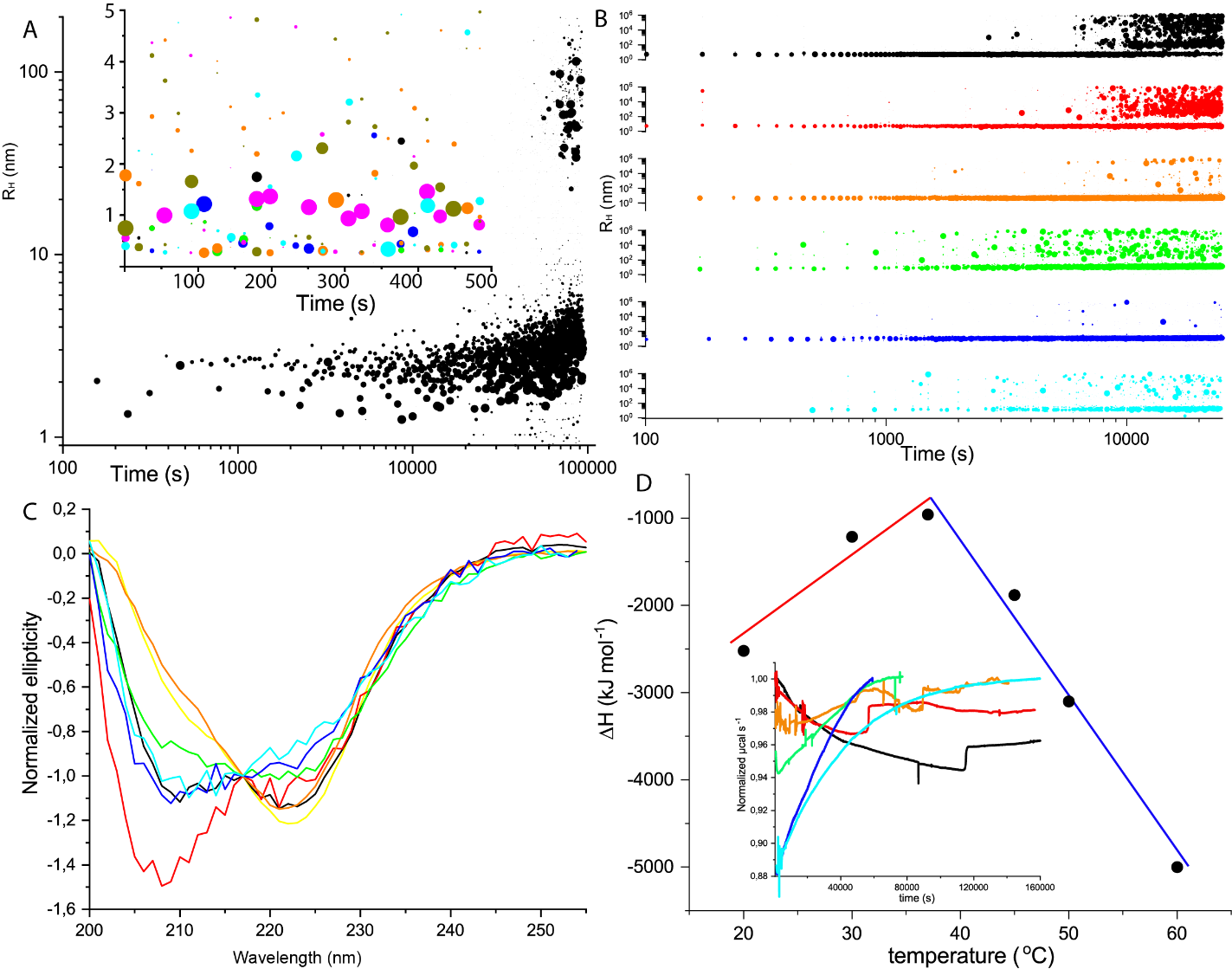


**Fig. S1**: (A) DLS measurement of a freshly prepared His_6_-tagged DISC1 C-region sample at 20 ^o^C. During the initial phase of the measurement, the sample has a R_H_ of 2.94 nm and aggrandizes to ~100 nm towards the end of the measurement. The inset figure illustrates determination of the minimum detectable concentration of the His_6_-tagged version at 20 °C, using various concentrations: 15 µM (olive green), 10 µM (orange), 7.5 µM (pink), 5 µM (cyan), 2.5 µM (blue), 1.25 µM (green) and 0.75 µM (black). To study the oligomerization propensity of the MBP fusion construct of the DISC1 C‑region, we performed (B) DLS and (C) CD measurements to follow changes in SSE at various temperatures, 20 ^o^C (black), 30 ^o^C (red), 37 ^o^C (orange), 45 ^o^C (green), 50 ^o^C (blue) and 60 ^o^C (cyan). One additional spectrum (yellow) in the CD profile depicts the freshly prepared sample. (D) Thermograms (inset) of 10 µM protein solutions recorded at 20 °C (black), 30 °C (red), 37 °C (orange), 45 °C (green), 50 °C (blue) and 60 °C (cyan), with corresponding enthalpy changes plotted in the main graph. ΔCp values of 94.3 and ‑179.8 kJ mol^‑1^ K^-1^ were derived from the slopes of the two segments using a linear fit.


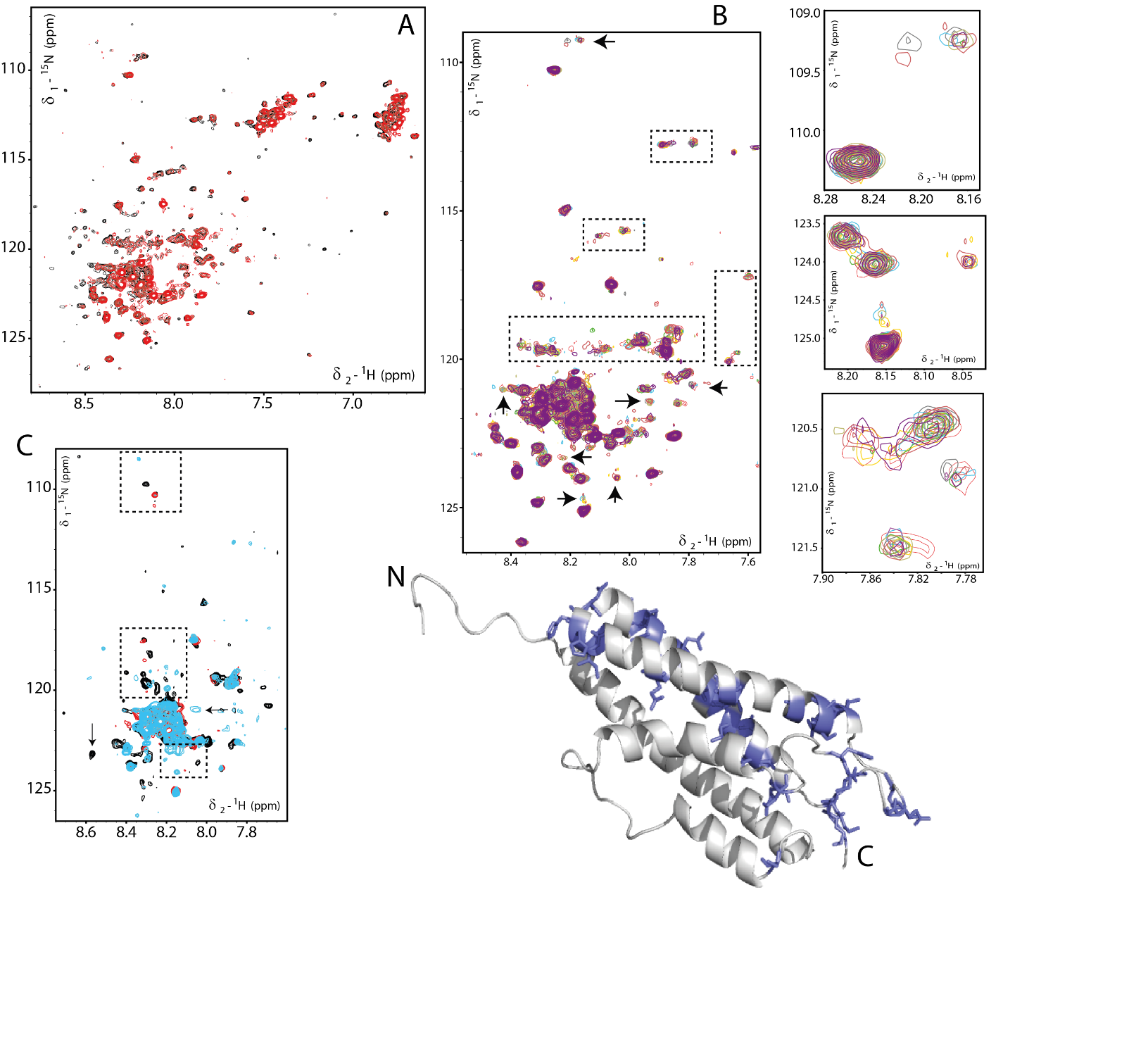


**Fig. S2**: (A) Solution NMR spectroscopy measurements of C-region DISC1 at protein concentrations, 400 µM (black) and 60 µM (red) describes spectra of oligomerized and/or unfolded protein; we expect to observe 166 backbone resonances for a monomeric folded protein. Spectra was processed with squared sine bell function of 2 (B) and (C) describes time dependent measurements at protein concentrations of 60 µM (sine bell function of 2) and 10 µM (processed using exponential line broadening factor of 10 Hz), respectively. The colour coding in the spectra depicts various time points in minutes; red (0-300), grey (300-600), green (600-900), yellow (900-1200), cyan (1200-1500), khaki (1500-1800), bronze (1800-2100) and purple (2100-2400).


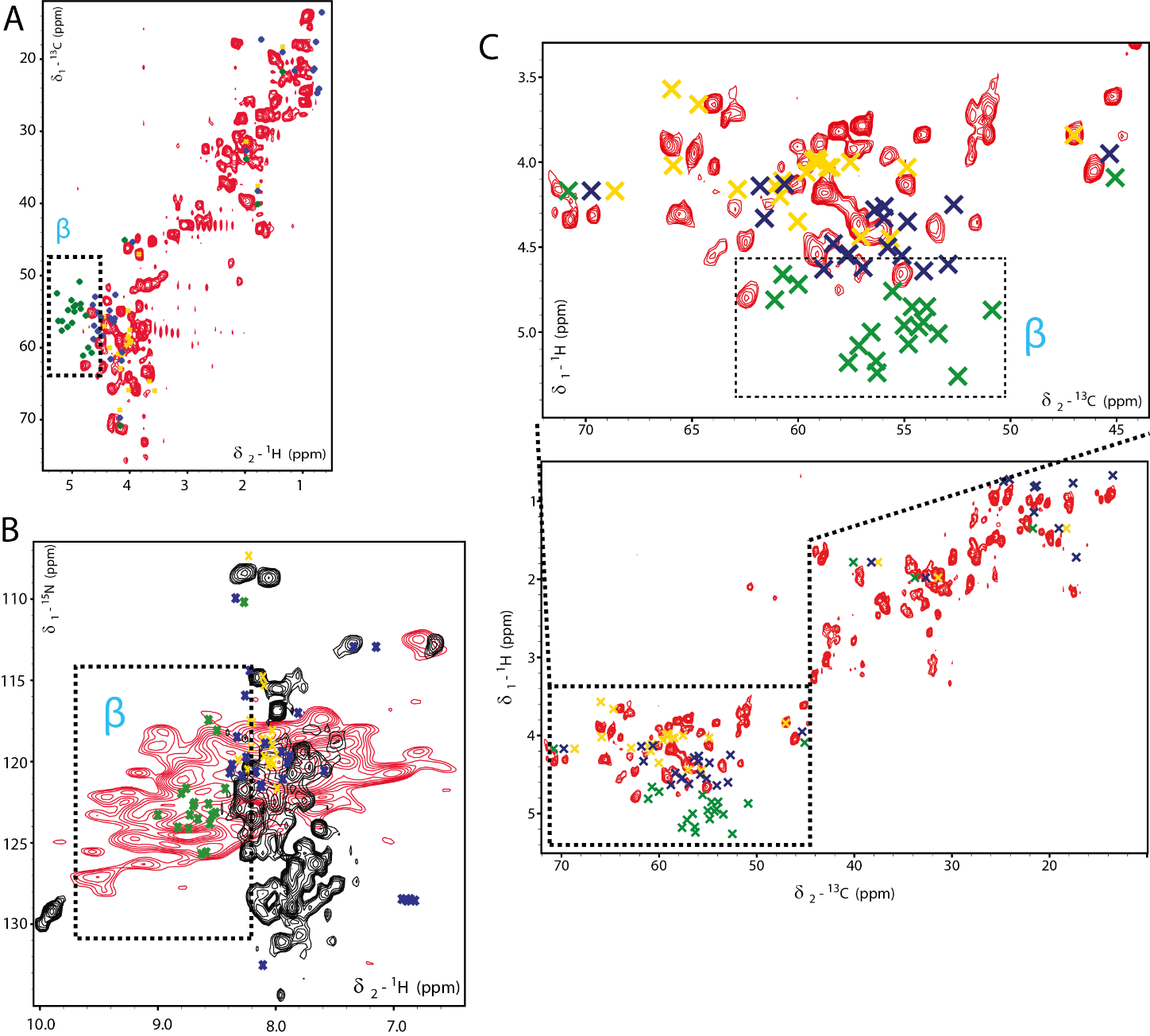


**Fig. S3**: (A) 2D ^1^H-^13^C-HSQC-MAS NMR spectrum of the solid sample; as in panels (B) and (C), annotations in yellow, blue and green represent average CS (Wang and Jardetzky, 2001 [**https://doi.org/10.1110/ps.3180102**](https://doi.org/10.1110/ps.3180102)) of residues in helices, coiled regions (RC) and strands, respectively. The box depicts the region where CS of residues in β-strands are expected. (B) 2D ^1^H-^15^N correlations on scalar (black) and dipolar (red) couplings, respectively at 30 kHz MAS displaying the predominance of CS arising from residues in β-strands in the rigid part of the protein. (C) 2D ^1^H‑^13^C‑INEPT based correlation spectrum was applied to the DISC1 C-region protein. The top panel displays a zoom-in of the backbone ^1^H-^13^C correlation; the boxed region with green cross-peaks are suggestive of expected signals from residues if present in β-sheets.


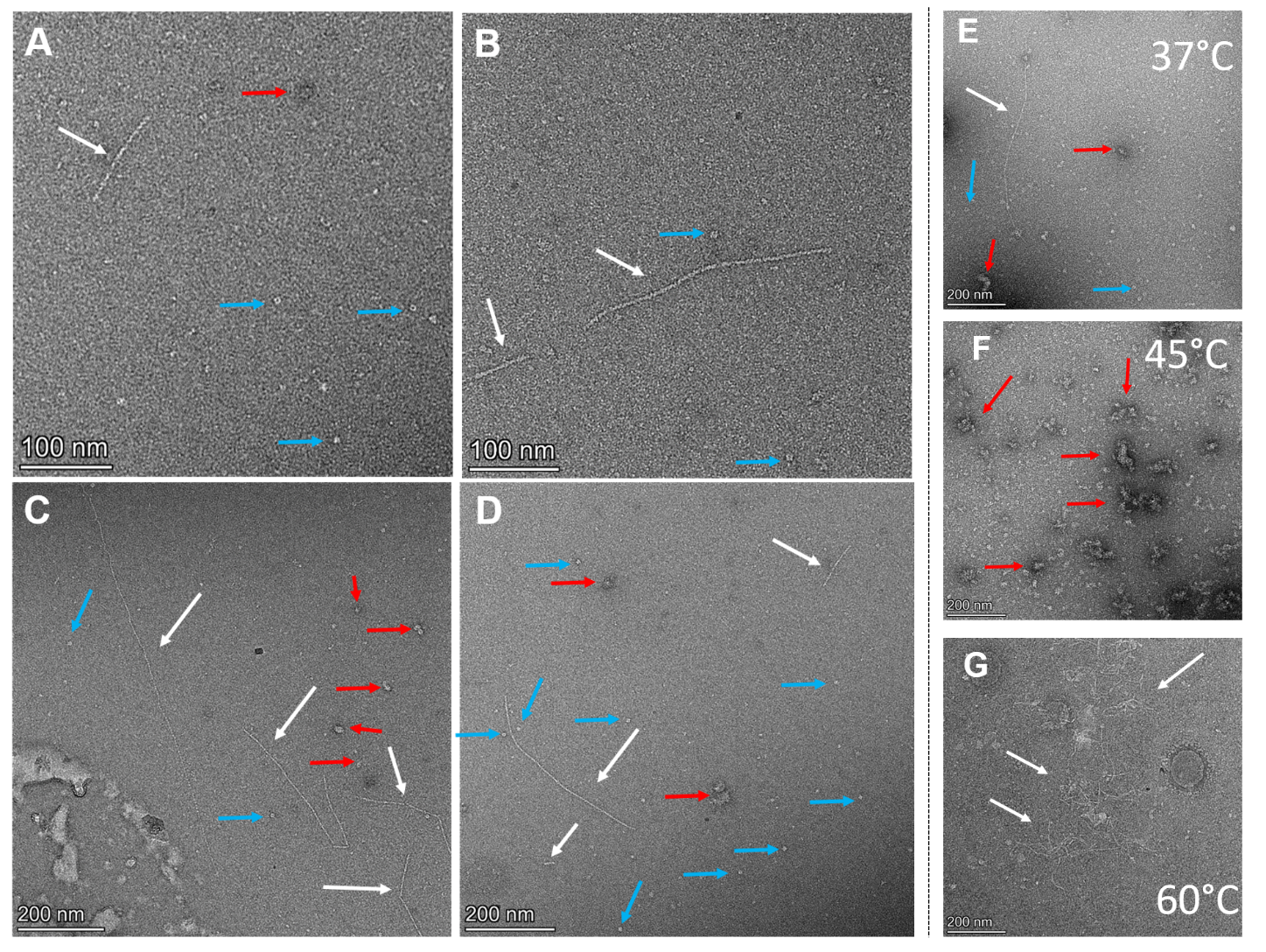


**Fig. S4**: Electron micrographs (A-D) of the DISC1 C-region predominantly shows 2 major populations comprising of the fibrils (white arrows) and oligomers (blue arrows). One can observe fibrils at various lengths ranging from 100 nm up to 800-1000 nm. We also observed amorphous aggregates (red arrows) in low abundance at temperatures of 37 ^o^C and lower. (D) Zoom-out image shown in Fig. 2C. (E-G) Negatively stained electron micrographs of the DISC1 C-region at various temperatures. Arrows coloured in white, red and blue point towards fibrils, oligomers and amorphous aggregates, respectively.


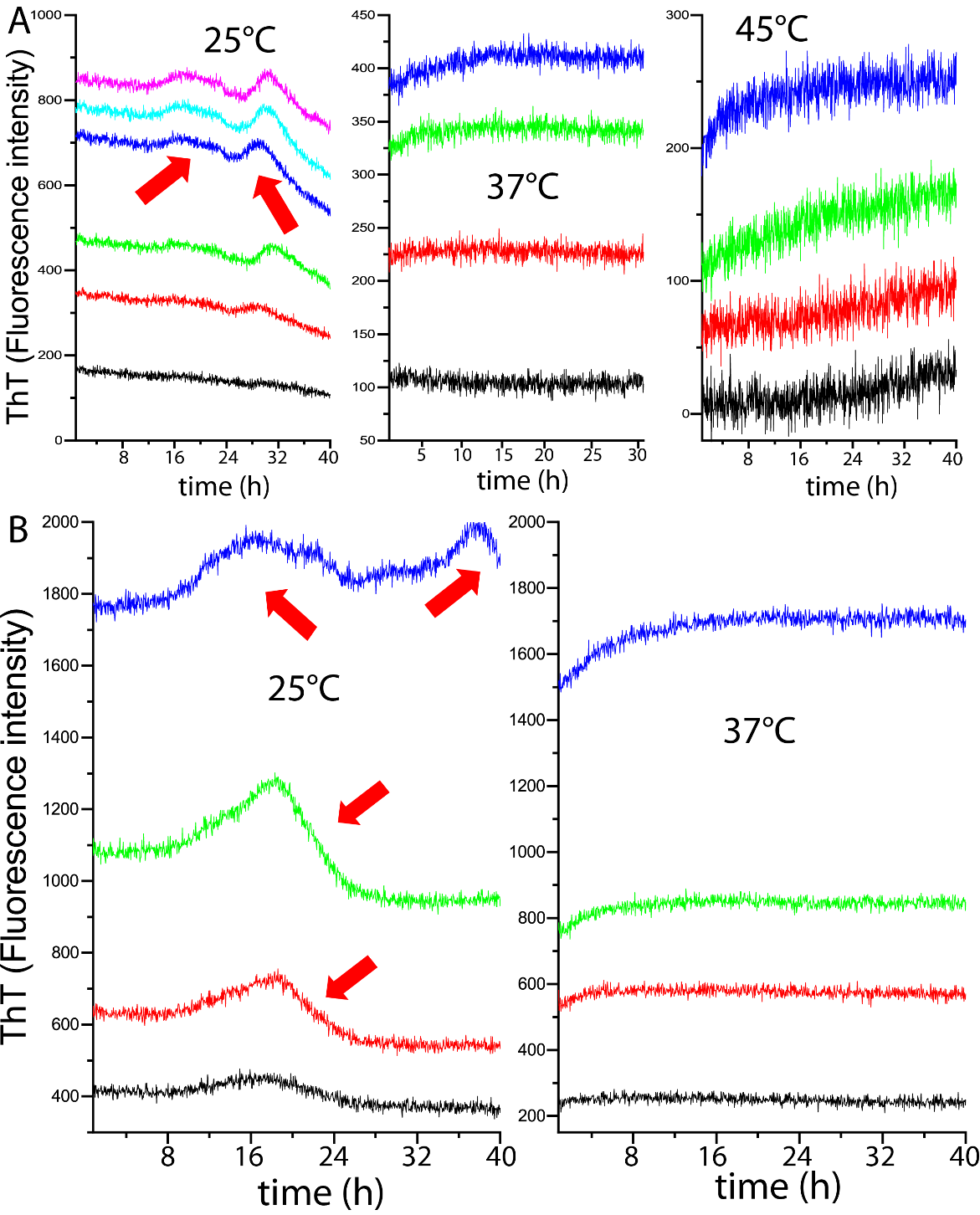


**Fig. S5**: (A) Temperature dependent effect of ThT fluorescence intensity at various DISC1 C‑region protein concentrations, 5 µM (black), 10 µM (red), 15 µM (green) and 20 µM (blue); 2 additional protein concentrations were tested at 25 µM (cyan) and 30 µM (pink).

(B) ThT fluorescence assay performed at 90 µM (blue), 45 µM (green), 22.5 µM (red) and 11.25 µM (black) at 25 °C and 37 °C. Oscillations between high and low intensities is most likely due to aggregation of the larger fibrils sticking to the sides of the plate during the measurement window followed by growth of another fibrillar segment(s).

**
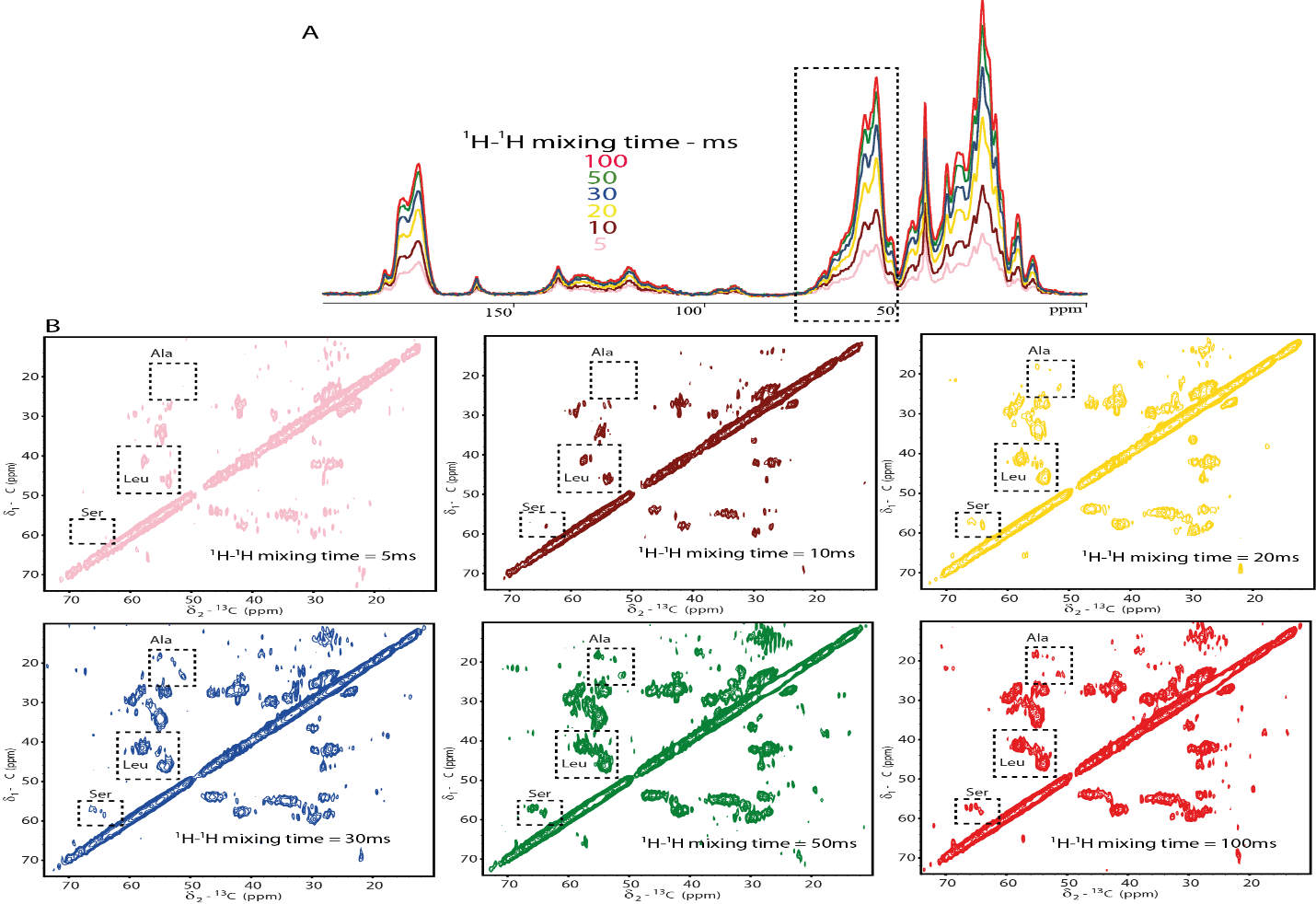
**

**Fig. S6**: (A) 1D ^13^C water build-up spectra with several with ^1^H-^1^H mixing times. The signal intensity in the Cα region (boxed) was plotted for the build-up curve depicted in Fig. 3E. (B) 2D ^13^C-^13^C correlation spectra with ^1^H-^1^H mixing times of 5 ms (pink), 10 ms (maroon), 20 ms (yellow), 30 ms (blue), 50 ms (green) and 100 ms (red), the boxed regions correspond to the signals from residues Ala, Leu and Ser that were used for the amino acid specific water build-up analysis depicted in Fig. 3E.


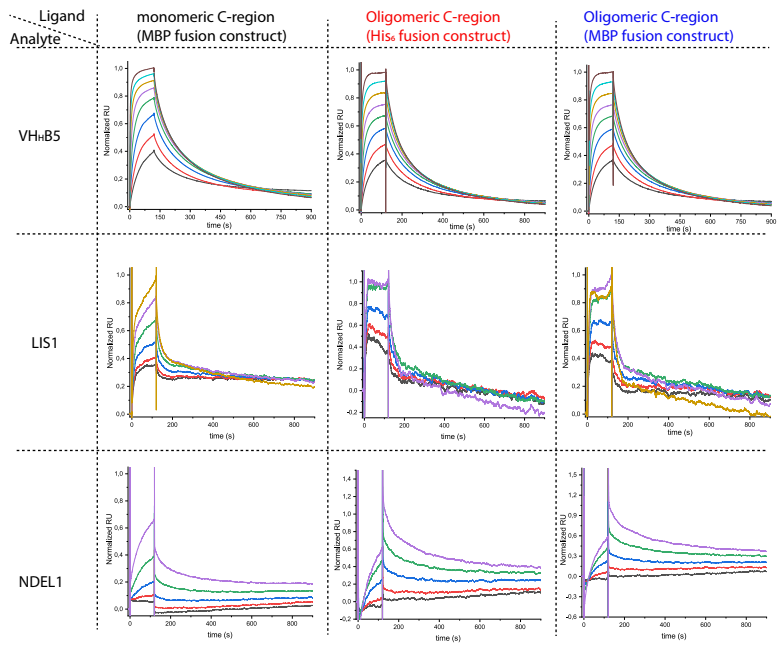


**Fig. S7**: (A) SPR sensorgrams featuring binding of V_H_H B5, LIS1 and NDEL1 with monomeric MBP‑C‑region, oligomeric His_6_-C-region and oligomeric MBP-C-region.


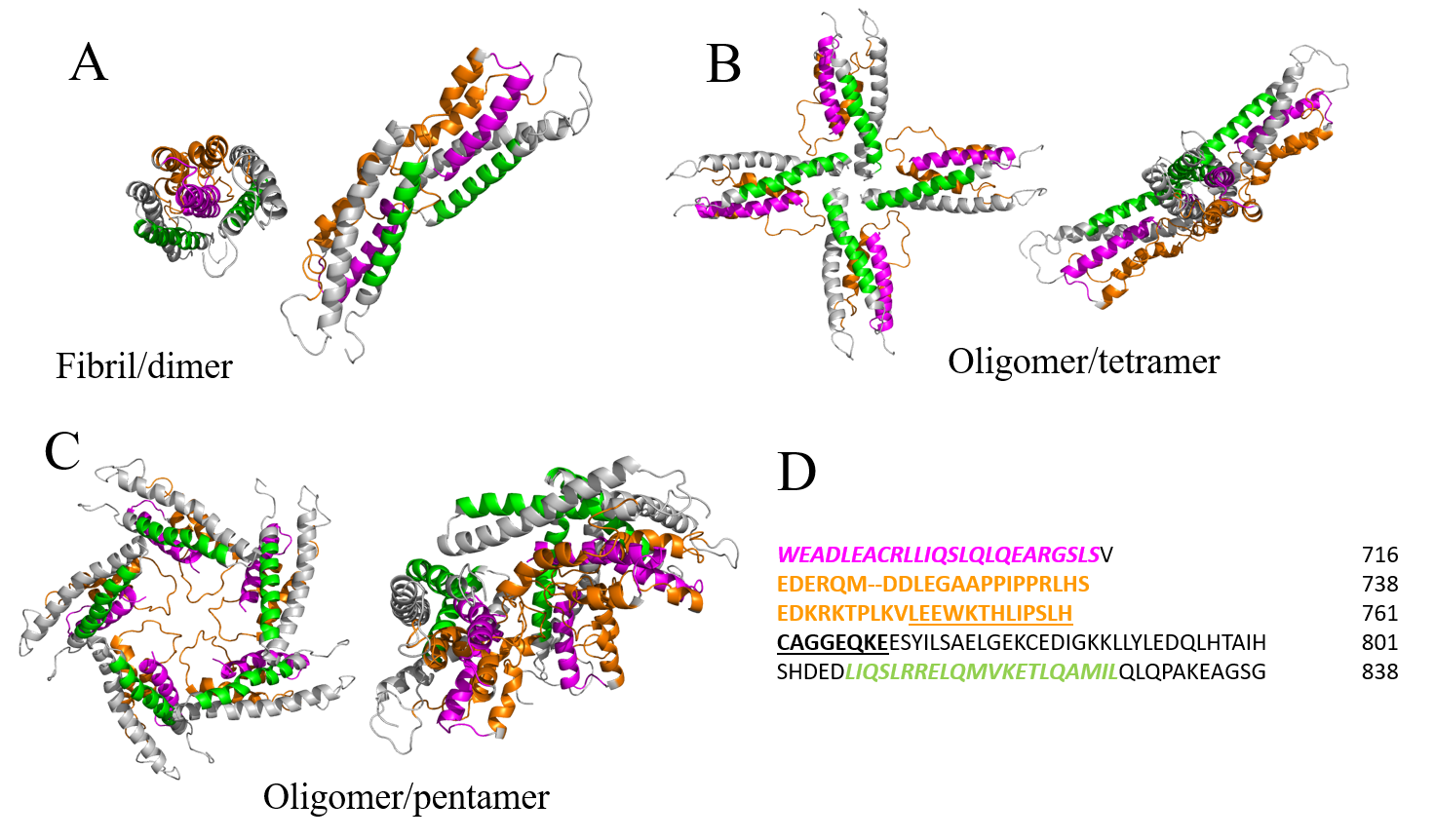


**Fig. S8**: Additional structural models (also see Fig. 4) of the DISC1 C‑region, which were generated using CLUSPRO2 and agree well with the experimental data. Fibrils (A) and oligomers (B and C) are colour coded as shown in the protein sequence in (D). Colours represent V_H_H B5 binding in magenta; (pseudo)repeat sequence in orange; underlined part describes residues in Δ22 and the green region represents the NDEL1 binding site.
